# Drift of Schottky Barrier Height in Phase Change Materials

**DOI:** 10.1021/acsnano.3c11019

**Published:** 2024-03-08

**Authors:** Rivka-Galya Nir-Harwood, Guy Cohen, Amlan Majumdar, Richard Haight, Emanuel Ber, Lynne Gignac, Efrat Ordan, Lishai Shoham, Yair Keller, Lior Kornblum, Eilam Yalon

**Affiliations:** † Viterbi Faculty of Electrical & Computer Engineering, Technion-Israel Institute of Technology, Haifa 32000, Israel; ‡ IBM Thomas J. Watson Research Center, Yorktown Heights, New York 10598, United States

**Keywords:** phase change memory, contact resistance, resistance drift, Schottky barrier height, thermionic emission

## Abstract

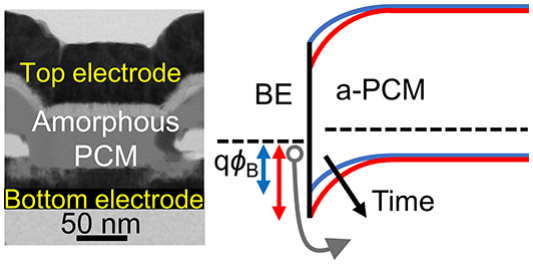

Phase-change memory (PCM) devices have great potential as multilevel memory cells and artificial synapses for neuromorphic computing hardware. However, their practical use is hampered by resistance drift, a phenomenon commonly attributed to structural relaxation or electronic mechanisms primarily in the context of bulk effects. In this study, we reevaluate the electrical manifestation of resistance drift in sub-100 nm Ge_2_Sb_2_Te_5_ (GST) PCM devices, focusing on the contributions of bulk vs interface effects. We employ a combination of measurement techniques to elucidate the current transport mechanism and the electrical manifestation of resistance drift. Our steady-state temperature-dependent measurements reveal that resistance in these devices is predominantly influenced by their electrical contacts, with conduction occurring through thermionic emission (Schottky) at the contacts. Additionally, temporal current–voltage characterization allows us to link the resistance drift to a time-dependent increase in the Schottky barrier height. These findings provide valuable insights, pinpointing the primary contributor to resistance drift in PCM devices: the Schottky barrier height for hole injection at the interface. This underscores the significance of contacts (interface) in the electrical manifestation of drift in PCM devices.

Phase change memory (PCM) is an excellent candidate for neuromorphic computing hardware because it can exhibit multiple intermediate resistance states,^1−7^ which is an important requirement for artificial synapses.^8−11^ Phase change materials, such as Ge_2_Sb_2_Te_5_ (GST), can reversibly switch between two main phases (states): polycrystalline (low resistance) and amorphous (high resistance), as shown schematically in Figure 1a. The phase transition is thermally induced by Joule heating, and the electrical resistance of the cell varies significantly with its phase, encoding the memory state. Yet, a key bottleneck for using PCM with multiple intermediate states for neuromorphic hardware is the increase in resistance with time, namely resistance drift,^12,13^ which can result in undesired synaptic weight change.^14−16^ This increase is most significant in the amorphous phase (a-GST), but it is also evident across intermediate states.^17^

**Figure 1:**
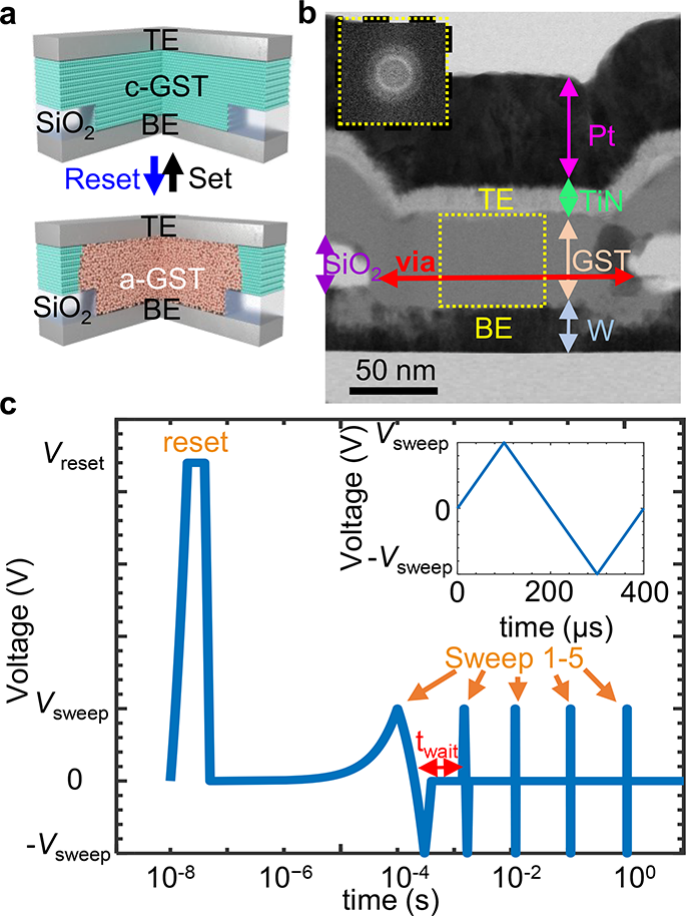
PCM device structure and measurement scheme. (a) Schematic illustration of our confined GST PCM device. (b) Bright field scanning transmission electron microscope (BF-STEM) image of our device in the amorphous phase. Via diameter is ∼150 nm and GST thickness is ∼50 nm. Inset shows a diffractogram from high resolution BF-STEM image of the area between the two electrodes in the amorphous phase. (c) Schematic of the measurement waveform, including a reset pulse followed by multiple read *I*–*V* sweeps with time intervals on a logarithmic scale. Inset shows a single sweep in linear scale.

Previous studies have attributed drift in a-GST to either structural relaxation,^18−22^ or purely electronic mechanisms,^23−25^ both referring mostly to bulk effects. Typically, the Poole–Frenkel (PF) model is employed to describe charge transport in a-GST.^26,27^ However, both our current work and previous research,^28−31^ indicate that electrical contacts dominate the resistance of sub-100 nm-long (lateral) or -thick (vertical) PCM devices. In this study, we employ a combination of techniques, including the transfer length method (TLM), the contact end-resistance method, temperature-dependent current–voltage (*I*–*V* ) measurements, and ultraviolet photoelectron spectroscopy (UPS), to determine the dominant current transport mechanism. Our findings reveal that conduction in our a-GST devices is governed by thermionic emission of holes at the contact (p-type). With this understanding, we perform temperature-dependent temporal *I*–*V* measurements to analyze resistance drift, focusing on the dominant conduction mechanism: emission over the Schottky barrier. We discover that the drift manifests itself as an increase in the Schottky barrier height (ϕ_B_) with time.

## Results and Discussion

### Time Dependent *I*–*V*

**Figure 2:**
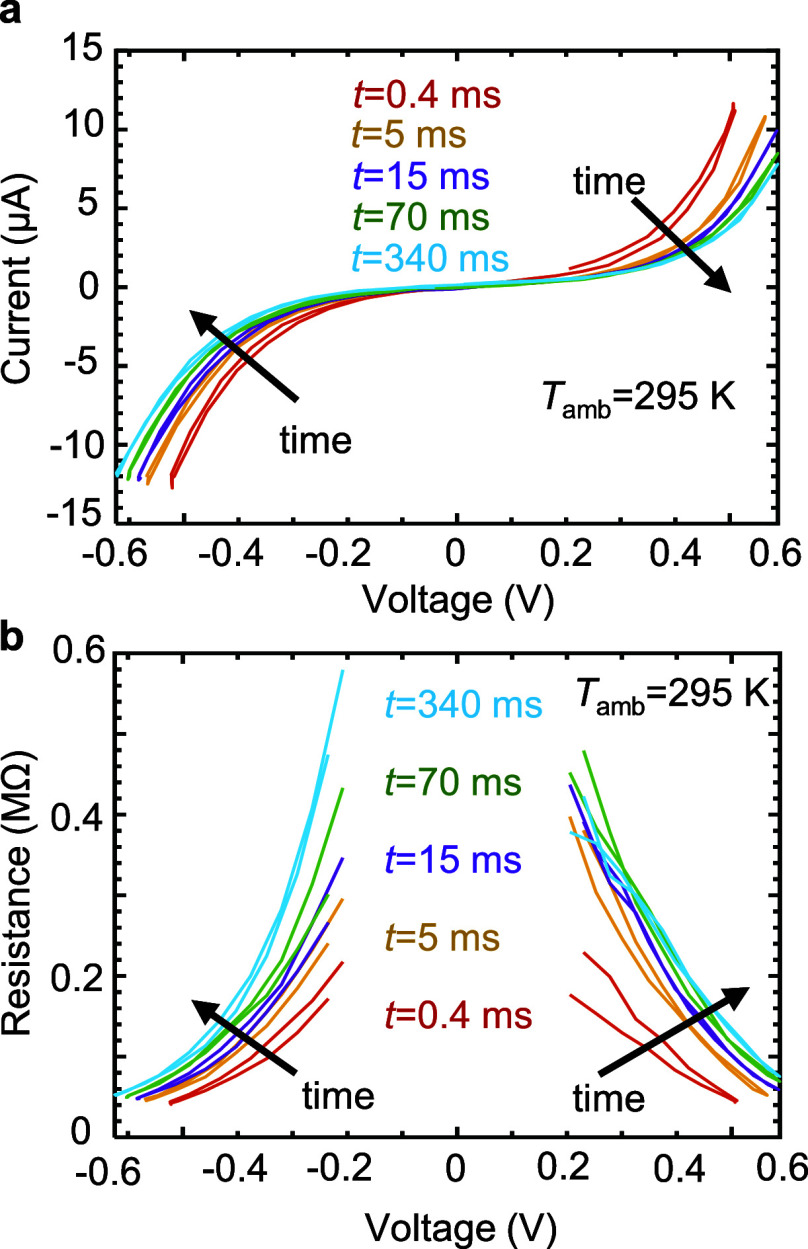
Time dependent *I*–*V* measurements. (a) *I*–*V* and (b) *R*–*V* characteristics in the amorphous phase at *T*_amb_ = 295 K, for varying drift time, following a reset pulse. Voltage is applied to the bottom electrode (BE). *I*–*V* is nonlinear and shows a drift with time. Resistance is defined by *R*(*t*) = *V*(*t*)/*I*(*t*).

We carried out a systematic study, including fast *I*–*V* sweeps to read the PCM resistance vs time following a reset pulse (resistance drift). Typically, PCM resistance is read at a single voltage, but here, we use *I*–*V* sweeps to uncover the current transport mechanism and explore the voltage dependence. We studied conventional PCM cells of confined GST (Figure 1a,b), fabricated as outlined in the Methods section, and performed a series of fast *I*–*V* sweeps. The sweeps were performed immediately after applying a reset pulse (Figure 1c), separated by time intervals on a logarithmic scale, for different ambient temperatures in the range *T*_amb_ = 80–295 K. Figure 2a shows a series of *I*–*V* measurements executed at different time intervals from the reset pulse. We observe nonlinear behavior, signifying the non-Ohmic nature of conduction in a-GST devices.^18,28,32^ In addition, the figure shows that the current at a given voltage decreases with time. Consequently, the resistance, defined as *R*(*t*) = *V*(*t*)/*I*(*t*), increases with time (Figure 2b). We show drift results for a device with via diameter of ∼150 nm and thickness of ∼50 nm at *T*_amb_ = 295 K, but other devices exhibited similar behavior in the measured temperature range.

### Voltage- and Temperature-Dependent Drift

**Figure 3:**
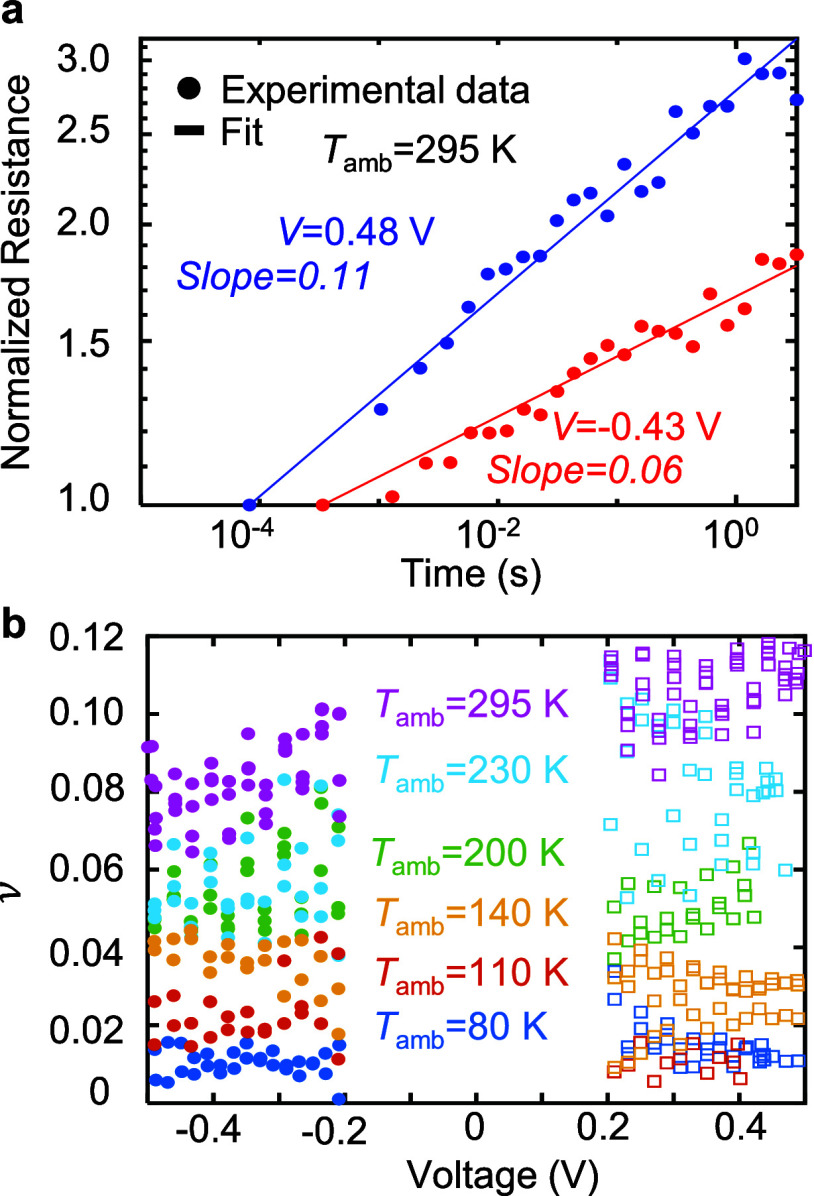
Voltage- and temperature-dependent drift coefficient. (a) Log–log plot of normalized resistance vs time for two representative read voltages (−0.43 V red, 0.48 V blue), to extract drift coefficient, ν (slope) according to *R*(*t*)/*R*_0_ = (*t*/*t*_0_)^ν^. (b) Fitted drift coefficient vs read voltage for different temperatures. The drift coefficient has a strong *T*-dependence and a less pronounced *V*-dependence.

Using the temporal *I*–*V* data, we evaluate the resistance drift voltage- and temperature-dependence. We obtain the drift coefficient ν according to the power law:^18^
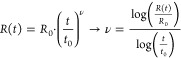
1where *R*_0_ and *t*_0_ are the resistance and time of the first measurement after reset (Figure 3a). Initial resistances were in the range *R*_0_ = 100–350 kΩ. Figure 3b shows that the drift coefficient has an appreciable temperature-dependence, increasing as *T*_amb_ increases, whereas the voltage-dependence is much less pronounced. This result is consistent with other reports in the literature.^33,34^ Furthermore, higher initial resistance results in a higher drift coefficient.^3,35,36^ This helps explain some anomalies in Figure 3b for which the drift coefficient is not higher at higher temperatures. For example, the drift coefficient at 80 K appears to be higher than the coefficient at 110 K for positive bias.

### Contact vs Bulk Resistance

Next, we sought to determine the dominant current transport mechanism in a-GST devices. These mechanisms could be categorized into two main groups: bulk dominated (Poole–Frenkel, hopping, space-charge, etc.) and contact dominated (thermionic emission, tunneling, etc.). Traditionally, the Poole–Frenkel mechanism has been used to describe conduction in a-GST.^26,27^ However, recent research suggests that contacts may play a more significant role.^28−31^ In this study our steady-state measurements lead to the conclusion that for sub-100 nm a-GST devices, the conduction is dominated by the contacts, and specifically the thermionic emission (Schottky) mechanism.^37^ These steady-state measurements were conducted on three different test structures of a-GST. Further details about the methods, sample differences, and limitations can be found in Supporting Information Section S1.

**Figure 4:**
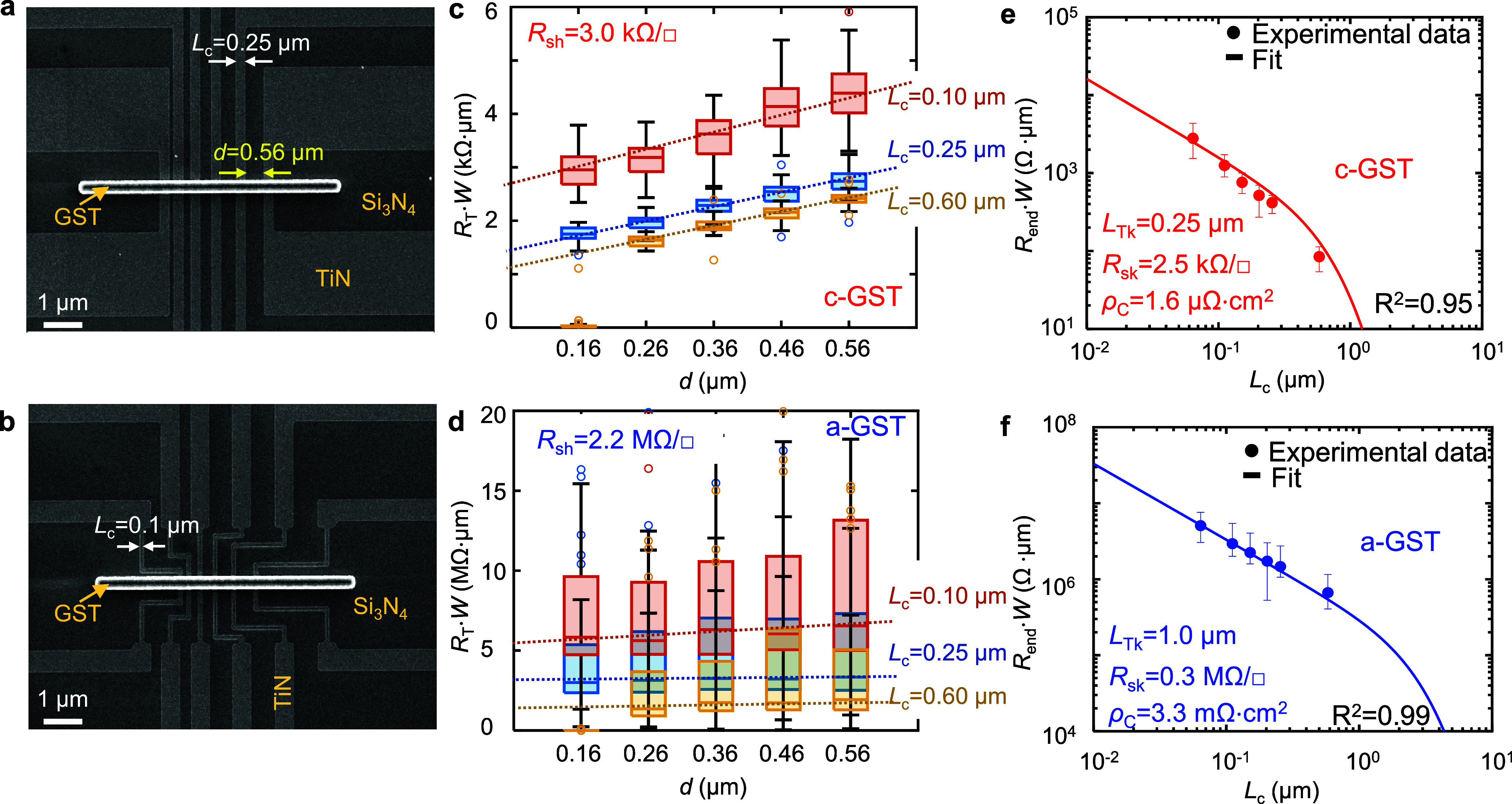
Transfer length method (TLM) measurements. Scanning electron microscope (SEM) images of the TLM test structures for contact lengths of (a) *L*_c_ = 0.25 μm and (b) *L*_c_ = 0.10 μm. Resistance width product (*R*_T_·*W)* as a function of spacing between contacts, *d* for (c) c-GST, and (d) a-GST. Plots show statistical analysis of 48 measured structures and include dashed lines as a guide for the eye. Contact-end resistance as a function of contact length *L*_C_ for (e) c-GST, and (f) a-GST. Dots represent median values and error bars the 85% confidence intervals. Results indicate that the resistance in both c-GST and a-GST is dominated by the contacts in sub-100 nm spacing.

In order to identify whether the conduction is bulk or contact limited, we use the well-known transfer length method.^38^Figure 4a,b show the test structures for which the contact lengths (*L*_c_) and contact spacings (*d*) are a few-hundred-nm, fabricated and measured as described in the Methods section. We extract the sheet resistance (slope) and the contact-front resistance (half the ordinate intercept) for both crystalline phase (c-GST) and the amorphous phase (Figure 4c,d), according to^38^
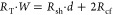
2where *R*_T_ is the total resistance, *W* is the GST width, *R*_sh_ is the sheet resistance, and *R*_cf_ is the contact-front resistance. For c-GST the sheet resistance is *R*_sh_ = 3.0 kΩ/□ (85% confidence intervals: 2.3–5.4 kΩ/□), and for a-GST *R*_sh_ = 2.2 MΩ/□ (85% confidence intervals: 0.3–11.8 MΩ/□). In both phases, the contact-front resistance is dominant, signifying the negligible contribution of the sheet resistance compared to the contact resistance. We also used contact-end measurements to evaluate the contact resistivity. Figure 4e,f show the contact-end resistance as a function of the contact length, and the fitted plot, according to^38^
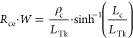
3Here *R*_ce_ is the contact-end resistance, ρ_c_ is the contact resistivity, and *L*_Tk_ is the transfer length on top of the contacts (*L*_Tk_ = (ρ_c_/*R*_sk_)^0.5^, where *R*_sk_ is the sheet resistance on top of the contacts). For c-GST the contact resistivity is ρ_c_ = 1.6 μΩ·cm^2^ (85% confidence intervals: 1.0–2.4 μΩ·cm^2^), and for a-GST ρ_c_ = 3.3 mΩ·cm^2^ (85% confidence intervals: 2.0–5.4 mΩ·cm^2^). A summary of the fitted resistivity values can be found in the first row of Table 1. The contact-front measurements and fits are shown in Supporting Figure S2, and even though they have a higher error, they corroborate the findings from the contact-end measurements, enhancing the robustness of the results. Additional data regarding the TLM measurements can be found in Supporting Information Section S2.

**Table 1:** GST Resistivity^a^

contact	a-GST measurement strategy	a-GST type	ρ^a^ (mΩ·cm) a-GST bulk resistivity	ρ^c^ (mΩ·cm) c-GST bulk resistivity	ρ_C_^a^ (mΩ·cm^2^) a-GST contact resistivity	ρ_C_^c^ (mΩ·cm^2^) c-GST contact resistivity	γ^a^ (μm) ρ_C_^a^/ρ^a^	γ^c^ (μm) ρ_C_^c^/ρ^c^
this work, GST-TiN	TLM	Ge ion implanted	22 × 10^3^	30	3.3	1.6 × 10^–3^	1.57	0.53
Savransky,^39^ GST-metal	varying GST thicknesses	melt-quenched	10 × 10^5^	20	N/A	0.3 × 10^–3^	N/A	0.15
Roy,^30^ GST-TiW	cross-bridge Kelvin resistor	as-deposited	80.5 × 10^4^	20	95	3.8 × 10^–3^	1.18	1.90
Huang,^28^ GST-TiN	modified TLM	as-deposited	54.5 × 10^3^	34	64	80 × 10^–3^	11.74	23.52
Shindo,^31^ GST-W	circular TLM	as-deposited	22 × 10^5^	48	32	14 × 10^–3^	0.15	2.91
Adnane,^40^ GST-W	4-point-probe	as-deposited	87 × 10^4^	75 (fcc)	N/A	N/A	N/A	N/A
				11 (hcp)				

a Resistivity of GST ρ, specific contact resistivity *ρ*_*c*_, and characteristic length at which bulk resistance equals contact resistance γ, in both crystalline and amorphous phases, compared with those in previous studies. Findings indicate that for 100 nm-long (lateral) or -thick (vertical) devices, in both phases, the resistance is dominated by the contacts.

To assess the contribution of contacts vs bulk to the total resistance, we define a characteristic length (for lateral transport) or thickness (for vertical transport) at which bulk resistance equals contact resistance as γ = ρ_C_/ρ (same as the dominance factor of contact resistivity, in Shindo et al.^31^). The values obtained from our measurements, along with a comparison to those from previous reports, are summarized in Table 1. As expected, our results are slightly different from previous reports which use as-deposited a-GST and not ion-implantation. However, all findings indicate that for 100 nm-long (lateral) or -thick (vertical) devices, in both phases, the resistance is dominated by the contacts.^28−31,39^ Our measurements suggest that in thin films, the change in device resistance during switching is primarily attributed to changes in contact resistance, which can differ by more than 3 orders of magnitude (ρ_C_^a^/ρ_C_^c^ > 10^3^).

### Thermionic Emission (Schottky)

**Figure 5:**
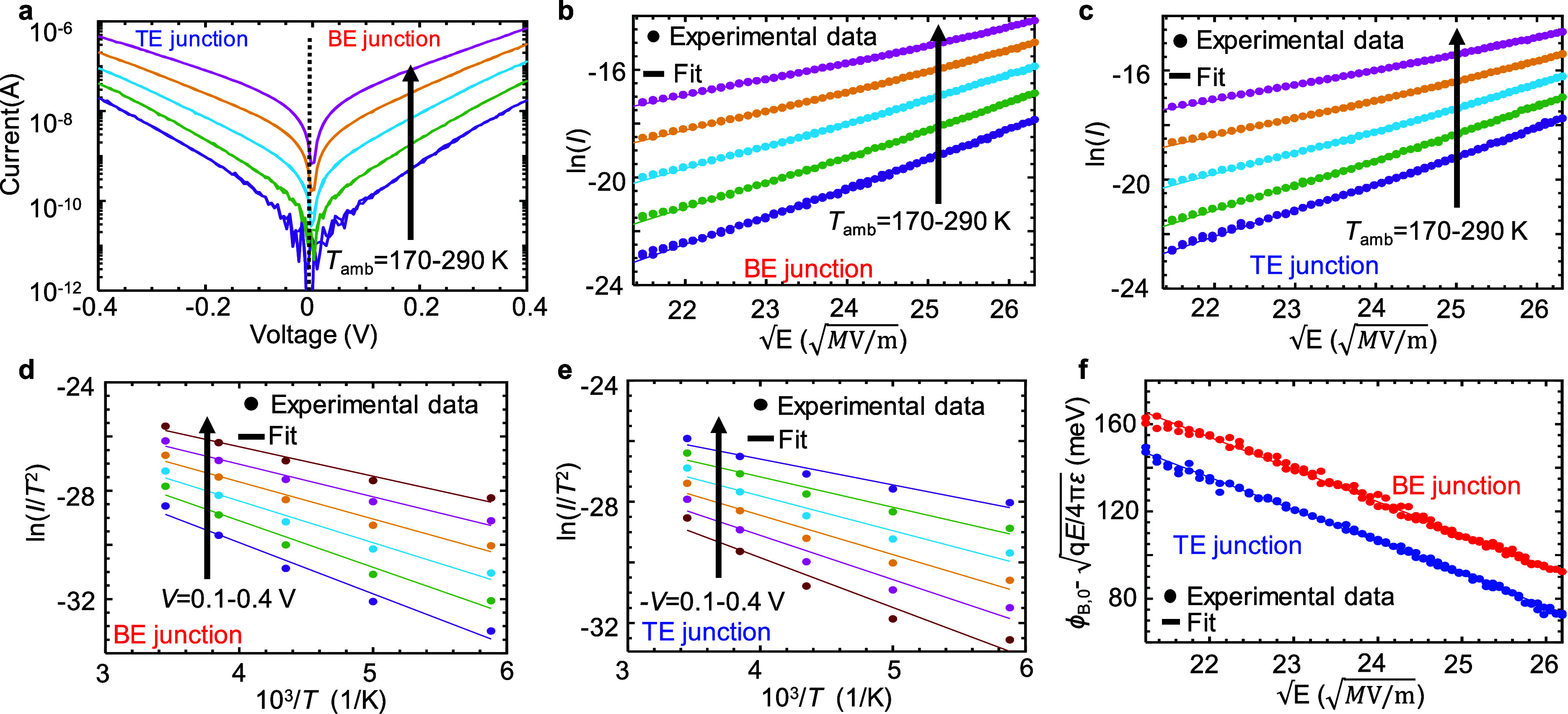
Thermionic emission (Schottky) fit. (a) *I*–*V* measurements for varying ambient temperatures. Via diameter of the confined area is ∼200 nm. Measured (markers) and fitted thermionic emission expression (lines) ln(*I*)–*E*^0.5^ for varying *T*_amb_ at (b) positive and (c) negative bias to bottom electrode (BE). Measured (markers) and fitted (lines): ln(*I*/*T*^*2*^) vs 10^3^/*T* for varying read voltages at (d) positive and (e) negative bias to BE. (f) Minimum barrier height extraction. The barrier, ϕ_B_, is obtained from the value at *V*_A_ = 0 V.

With the understanding that the contacts dominate the resistance, we seek to identify the conduction mechanism in the confined a-GST devices (Figure 1a,b). We performed steady state *I*–*V* measurements on the confined devices in the amorphous phase, at different ambient temperatures (Figure 5a). The thermionic emission mechanism (i.e., a reversed biased Schottky junction with image force barrier lowering) is the most appropriate contact dominated mechanism to describe our results (Figure 5b,c),^41−43^ and is given by^43^
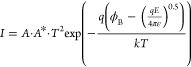
4where *A* is the contact area, A* is the Richardson constant, *T* is temperature in K, *ϕ*_B_ is the barrier height, *E* is the electric field, and ε is the dielectric constant. For clarification, in back-to-back Schottky diodes, the polarity of the voltage determines which junction is in reverse bias (top or bottom interface) and limits the conduction, as marked in Figure 5a.

Traditionally, the Poole–Frenkel (PF) conduction mechanism is used to describe the current transport in a-GST,^26^ due to reasonable *I*–*V* fitting and its symmetrical behavior. As both thermionic emission and PF are thermionic effects for which the current is exponentially dependent on *E*^0.5^, it could be difficult to distinguish between the two. Therefore, basic *I*–*V* analysis is insufficient for pinpointing the conduction mechanism. The TLM results, which show that the contacts dominate the resistance, establish that thermionic emission governs transport in sub-100 nm devices. Furthermore, the symmetrical *I*–*V* behavior has led previous work to reject the thermionic emission model.^44^ Yet, as a-GST has a high impurity density, it is very likely that high density of interface traps dictates the barrier height at the interface (i.e., Fermi level pinning),^45^ and only a slight dependence on the electrode material work function is expected.^46^ Consequently, the extracted barrier heights for the TE and BE junctions are similar, as detailed below, and a nearly symmetrical *I*–*V* behavior is observed.

We estimate the electric field in the proximity of the interface using the depletion approximation, given by^45,47^
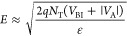
5where *N*_T_ is the ionized trap density, *V*_BI_ is the built-in voltage, and *V*_A_ is the applied voltage. We do not assume uniform field due to the nonzero built-in field and the nonlinear *I*–*V* characteristics.

Using the method described in Yeargan et al.,^48^ we obtain the plot slopes of ln(*I*/*T*^2^) vs 1/*T* (Figure 5d,e) and the change in barrier height due to an electric field, known as image force barrier lowering (Figure 5f). The minimum barrier height, extracted from Figure 5f from the value at *V*_A_ = 0,^38^ is ∼210 and 190 meV for the tungsten BE contact and TiN TE contact, respectively. More details on the thermionic emission fit are available in Supporting Information Section S3.

**Figure 6:**
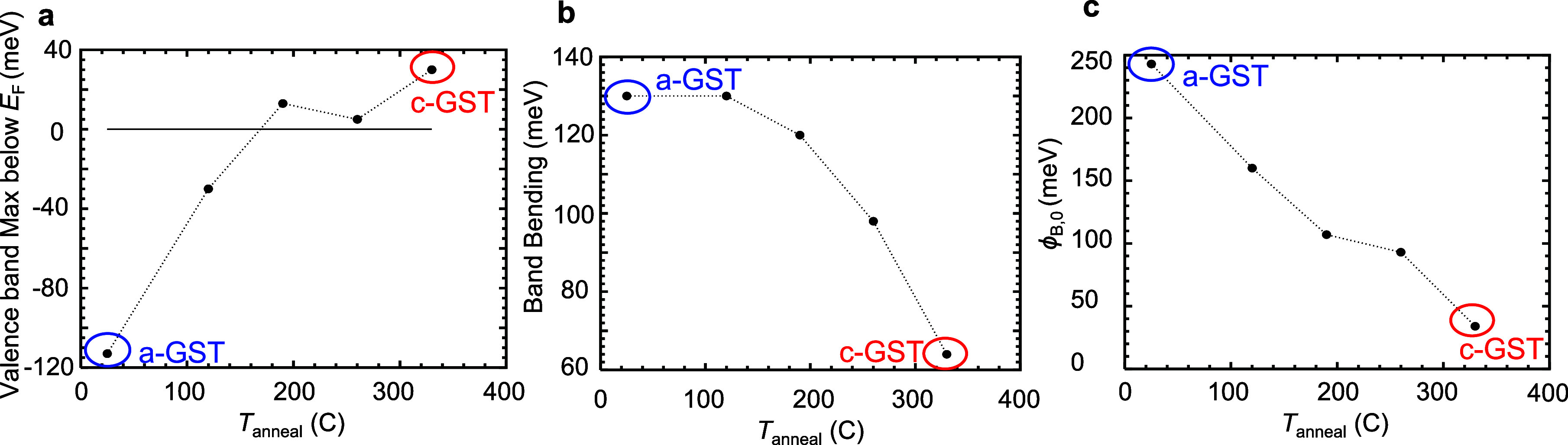
Ultraviolet photoelectron spectroscopy (UPS) measurements. (a) Valence band max below *E*_F_ vs anneal temperature. (b) Band bending vs anneal temperature. (c) Barrier height vs anneal temperature.

Additionally, we performed pump/probe ultraviolet photoelectron spectroscopy (UPS) measurements on as-deposited a-GST. Results show that a-GST behaves as a p-type semiconductor (Figure 6a), in agreement with previous work.^40,46,49,50^ For a-GST, the built-in voltage, *V*_BI_, and the barrier height, ϕ_B,0_, are approximately 130 and 240 meV, respectively (Figure 6b,c). These results are consistent with the steady state *I*–*V* measurements and the thermionic emission model. More details of the UPS measurements can be found in Supporting Information Section S4.

For Multilevel PCM implementations, the conventional understanding is that intermediate resistance states could be achieved using varying amorphous volumes.^19,51^ Our finding that the thermionic emission conduction dominates the resistance in nanoscale devices indicates that we should also consider the varying interface areas of the amorphous material as the source of intermediate resistance states. Both top and bottom interfaces can play a role, and a barrier may exist not only between the metal and a-GST but also between crystalline and amorphous GST.^52^ The dominant interface depends on the device geometry and the polarity of the applied voltage. More information can be found in Supporting Information Section S5.

### Drift in Schottky Barrier Height

**Figure 7:**
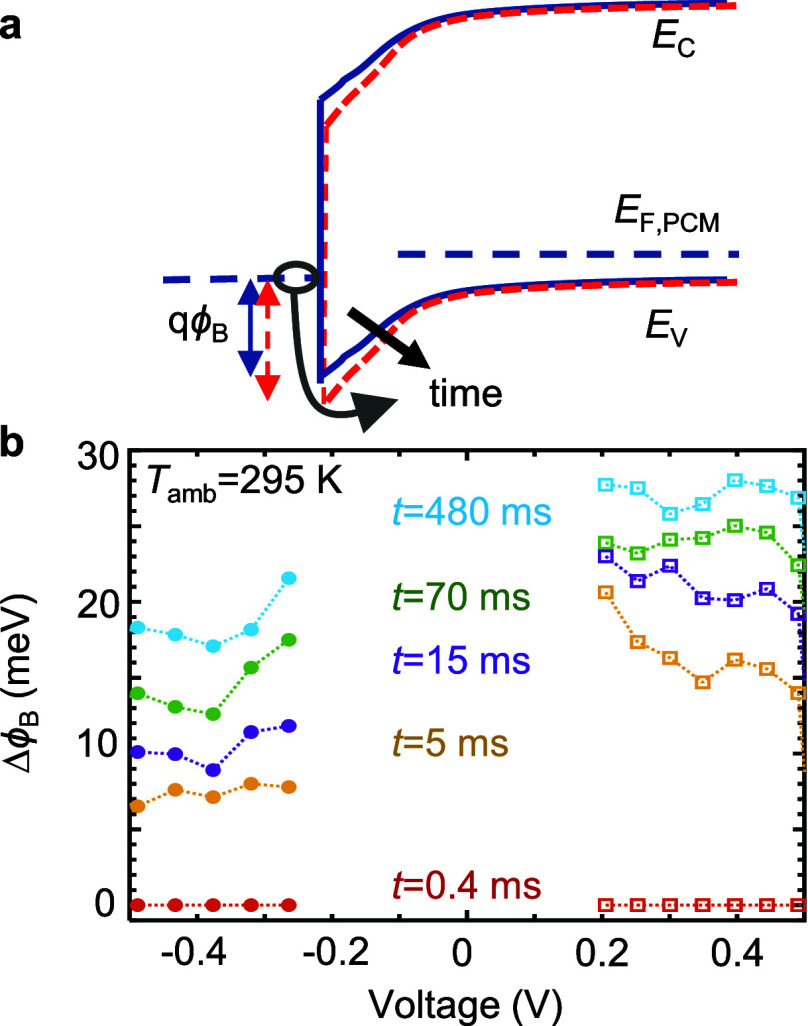
Extraction of drift in the Schottky barrier height. (a) Schematic band diagram with barrier height drift. The energy barrier height for hole injection to GST increases with time (drift). GST is a p-type semiconductor. (b) Extraction of the change in barrier height for varying drift times following a reset pulse according to eq 7.

Determining the conduction mechanism as a thermionic emission holds the key to understanding the electrical manifestation of the drift phenomenon in PCM devices. With this understanding we return to the temporal *I*–*V* measurements shown in Figure 2, pinpointing the drift to a physical variable, the contact barrier height for hole injection, as illustrated in Figure 7a. Thus, we extract the drift in barrier height (Figure 7b) by performing the following operations on the time dependent *I*–*V* measurements:
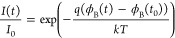
6
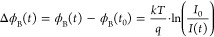
7where *I*_0_ and *t*_0_ are the current and time of the first measurement after reset. More information can be found in Supporting Information Section S6.

**Figure 8:**
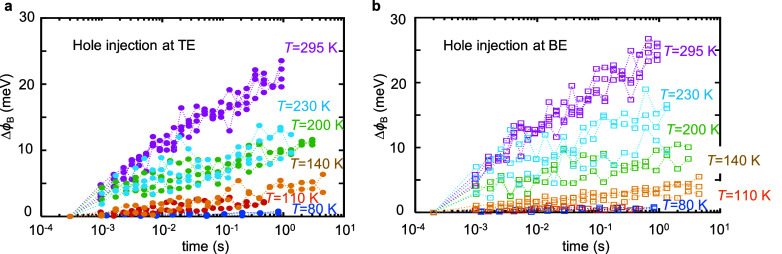
Drift in the Schottky barrier height. Extracted change in barrier height (Δϕ_B_) for hole injection to GST with time (drift) for varying temperatures at (a) TE and (b) BE. ϕ_B_ drift becomes more prominent at higher temperatures. However, current or resistance drift is observed even at low temperature due to the exponential dependence of the thermionic emission current on the barrier height. This result shows that the electrical manifestation of drift in the PCM is dominated by the interface (contact).

The average drift of the barrier height with time for all measured *T*_amb_ values is summarized in Figure 8. The barrier height drifts in the range of ∼1–30 meV for 80–295 K. These results are consistent with the power law behavior of PCM drift, expressed in eq 1. Combining the exponential dependence of the current on an energy barrier (log(*R*) ∼ *q*ϕ_B_/(*kT*)), thermionic mechanism) with our observation of a change in barrier height that is proportional to log(*t*), results in a direct relation between log(*R*) and log(*t*). Moreover, the current is exponentially dependent on the barrier height for different temperatures, explaining the strong temperature dependence of the drift coefficient. Our results show that the electrical manifestation of drift in PCM is dominated by the contacts.

To clarify, our results do not directly reveal the origin of the drift but rather the electrical manifestation. However, our findings can offer insights into previously unexplained experiments, particularly those related to the behavior of the drift coefficient at low temperatures and its sensitivity to illumination.^53^ The observation of resistance drift at low temperatures can be explained by the exponential relationship between the current and barrier height. Even when structural relaxation is significantly suppressed at low temperatures, a minor alteration in barrier height can lead to noticeable changes in measured resistance owing to the exponential characteristics of Schottky behavior. In the context of electron energy, previous research has suggested that drift could be attributed to a widening of the band gap over time,^13^ or changes in midgap states, such as charge traps.^18^ Both of these factors could potentially impact the barrier height, which, in turn, undergoes drift over time. In summary, our findings align with the structural relaxation explanation, yet they do not rule out a purely electronic origin of the drift.

## Conclusion

In conclusion, our work shows that the electrical manifestation of resistance drift in thin amorphous films is dominated by the contacts. We utilized a combination of techniques, including the steady-state transfer length method, contact-end measurements, temperature-dependent current–voltage (*I*–*V* ) characteristics, and ultraviolet photoelectron spectroscopy. Our findings reveal that the dominant current transport mechanism in amorphous thin GST-based phase change devices is thermionic emission (Schottky) of holes at the metal–semiconductor interface. Furthermore, our temporal temperature-dependent *I*–*V* sweeps indicate that drift in the PCM is reflected by an increase in the Schottky barrier height over time. We observed a strong temperature-dependence of the drift coefficient and a weak voltage-dependence in confined PCM devices with 50 nm thick GST. Our findings underscore the critical role of contacts in the evaluation of thin PCM devices, and enable a better understanding of drift, ultimately mitigating it, making PCM technology viable for neuromorphic applications.

## Methods

### Device Fabrication

The bright field scanning transmission electron microscope (BF-STEM) cross-section image of a confined PCM cell is shown in Figure 1b, and the devices were fabricated as follows.^54^ First, tungsten (W) was evaporated, patterned, and etched to form the bottom electrode (BE). Next, SiO_*x*_ was deposited using plasma enhanced chemical vapor deposition (PECVD) and the confined vias were patterned using electron-beam lithography. The BE was Ar sputter-cleaned to remove oxidation and prevent filament formation, followed by GST and TiN sputter deposition, all without breaking a vacuum. The top electrode (TE) was completed with additional TiN/Pt, and patterned by lift-off. Vias diameter ranges from 125 to 170 nm, and GST thickness is ∼50 nm.

The TLM structure shown in the scanning electron microscope (SEM) images in Figure 4a,b were fabricated as outlined: Planarized TiN contacts were formed by etching trenches in Si_3_N_4_, sputter-depositing TiN and planarizing the TiN by chemical mechanical polishing (CMP) such that the TiN surface is flashed with the Si_3_N_4_ surface. Argon (Ar) sputtering was used to clean the surface of the TiN contacts, which was followed by GST deposition without breaking the vacuum. This ensures a clean GST/TiN interface. The GST was annealed to form c-GST and was capped with Si_3_N_4_ film. Optical lithography followed by reactive ion etching was used to patterned the c-GST into bars, and the device was further encapsulated to prevent oxidation.

### Characterization

Electrical characterization of the confined PCM cells was carried out with a Keysight B1500 semiconductor parameter analyzer (SPA) in a JANIS probe station under vacuum conditions (<10^–4^ Torr) at temperatures ranging from room temperature (near 300 K) to 80 K.

The fast pulses were generated by a Waveform Generator/Fast Measurement Unit (WGFMU) which can generate arbitrary waveforms, such as the one depicted in Figure 1c. The system has an inherent trade-off between the measurement speed and the minimum current measured. Therefore, in order to read the high resistances, ranging from 100 to 350 kΩ, we performed 400 μs *I*–*V* sweeps (*t*_sweep_). We executed approximately 20–25 consecutive sweeps, and the time between consecutive sweeps was multiplied by ∼1.5–2 (*t*_multiply_). The time between *I*–*V* sweeps (*t*_wait_, marked in red in Figure 1c) can then be calculated using eq 8 for sweep number *n*:
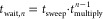
8During read operations, the highest measured voltage was below the threshold voltage in order to prevent destructive read.

BF-STEM images (Figure 1b) were done on a device from the same die as the device for which the temporal measurements were performed and with a similar via size. This device was reset to high resistance (1.5 MΩ) and then imaged a few weeks later. A top-down SEM image located the cross-section of the confined PCM cell for the focused ion beam (FIB) cut for the BF-STEM. Energy dispersive X-ray spectroscopy (EDX) was used to determine the elemental structure of the cross-section. The lack of diffraction patterns in the inset of Figure 1b suggests the absence of crystallinity, implying that the area between the two electrodes is amorphous. More images and details can be found in Supporting Information Section S7.

The TLM measurements for the crystalline and amorphous GST phases were performed on the same test sites (identical sizes). Initially the GST was in the crystalline phase. After measuring this phase, c-GST was amorphized by ion implantation of Ge.

Previous work has shown that by adjusting the ion dose implanted into c-GST the properties of the amorphized GST (such as the optical properties) can be tuned to match those of melt-quench a-GST.^55,56^ The electrical measurements on the amorphized GST were performed a few days after amorphization on a time span of less than 1 h, so the drift effects are negligible.

Similarly, the steady state *I*–*V* measurements were carried out a week after resetting the device to 500 kΩ to eliminate the influence of drift during data collection.

## Data Availability

The data that support this study are available from the corresponding author upon request. Other correspondence and requests for materials should be addressed to E. Yalon.

## Supporting Information Available

Comparison between methods and samples; Transfer length method, additional data; Steady state *I*–*V* measurements, additional data; Ultraviolet photoelectron spectroscopy, additional data; Interface dominated multilevel conduction in mushroom-type cells; Schottky barrier height drift, additional data; Transmission electron microscopy images (PDF)
