# Supplementary material for: Drift of Schottky Barrier Height in Phase Change Materials
Source: ACS Nano. 2024 Mar 8;18(11):8029–37. doi: 10.1021/acsnano.3c11019 (PMC10958591; doi:10.1021/acsnano.3c11019)
Supplement: Supplementary file 1 — nn3c11019_si_001.pdf [file nn3c11019_si_001.pdf]

Supporting Information

Drift of Schottky Barrier Height in Phase Change Materials

Rivka-Galya Nir-Harwood[1], Guy Cohen[2], Amlan Majumdar[2], Richard Haight[2], Emanuel Ber[1], Lynne Gignac[2], Efrat Ordan[1], Lishai Shoham[1], Yair Keller[1], Lior Kornblum[1], and Eilam Yalon[1*]

[1]*Viterbi Faculty of Electrical & Computer Engineering, Technion-Israel Institute of Technology, Haifa 32000, Israel*
[2]*IBM Thomas J. Watson Research Center, Yorktown Heights, New York 10598, United States*

[*]*E-mail:* eilamy@technion.ac.il

Contents

S1. Comparison between methods and samples 2
S2. Transfer length method, additional data 2
S3. Steady state *I-V* measurements, additional data 3
S4. Ultraviolet photoelectron spectroscopy, additional data 5
S5. Interface dominated multi-level conduction in mushroom-type cells 7
S6. Schottky barrier height drift, additional data 8
S7. Transmission electron microscopy images 9

S1. Comparison between methods and samples

In this work, we show that the dominant current transport mechanism in thin amorphous GST (a-GST) films is the thermionic emission (Schottky) at the contacts. For this, we employ three steady state measurements: the transfer length method (TLM) with Ge ion implanted a-GST, *I-V* measurements of melt-quench a-GST, and ultraviolet photoelectron spectroscopy (UPS) with as deposited a-GST. Even though crystallization kinetics may vary between the different amorphized samples,[1] the ion implantation achieves properties very similar to the melt-quench operation.[2] Moreover, it has also been shown that after ion implantation, properties such as the X-ray powder diffraction (XRD) spectrum and the electrical resistivity, are comparable to those measured in as deposited amorphous films.[3] Therefore, we assume that the electrical properties of the three types of a-GST used in the different measurements are similar, and ascertain the conduction mechanism in thin film a-GST.

For the TLM measurements, we used structures with horizontally separated contacts and under the GST film, with the assumption that the conduction is isotropic. A vertical structure, such as the one found in Savransky *et al.*,[4] which requires fabricating GST with varying thicknesses could result in an error in the measurement, because different material deposition (thickness) could lead to different properties, making the TLM vertical structure irrelevant.

S2. Transfer length method, additional data

The TLM method is used to show that the contacts dominate the resistance for both the amorphous and crystalline phases in thin GST films. The contact-front measurements and fits, shown in Supporting Figure S1, confirm the findings from the contact-end measurements, shown in Figure 4, strengthening the validity of the results. The contact-front measurement plots are fitted according to:[5]

$$R_{cf} \cdot W = \frac{\rho_c}{L_{Tk}} \cdot coth\left(\frac{L_c}{L_{Tk}}\right)$$

where R_{cf} is the contact-front resistance, W is the GST width, ρ_c is the contact resistivity, L_{Tk} the transfer length on top of the contacts ($L_{Tk}=(\rho_c/R_{sk})^{0.5}$, R_{sk} is the sheet resistance on top of the contacts), and L_C the contact length. For the c-GST ρ_c=1.4 μΩ·cm^2 (85% confidence intervals: 0.9–1.9 μΩ·cm^2), and for the a-GST ρ_c=2.9 mΩ·cm^2 (85% confidence intervals: 1.8-5.8 mΩ·cm^2). As

mentioned in the main text, contact-front measurements were dominated by the contact resistance compared to the sheet resistance. We note that the sheet resistance on top of the contacts (R_{sk}) and between the contacts (R_{sh}) are not identical. For c-GST R_{sh}=3.0 kΩ/□ while R_{sk}=2.5 kΩ/□, and for a-GST R_{sh}=2.2 MΩ/□ while R_{sk}=0.3 MΩ/□. It has been claimed that proximity to the metal can result in a higher conductivity of the semiconductor.[6]

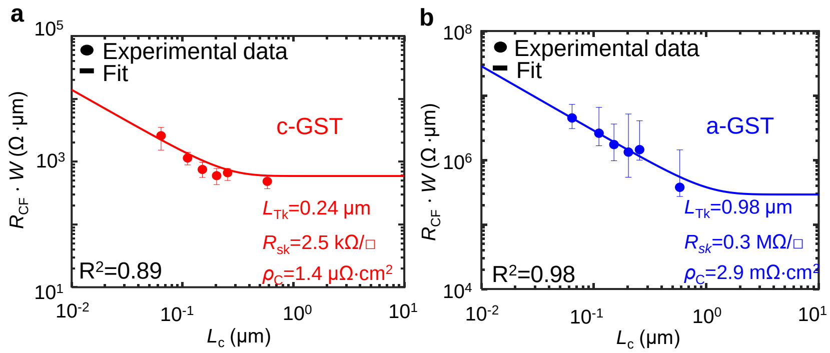

Fig. S1. Contact-front width product as a function of contact length for (a) c-GST (b) a-GST. Dots represent median value and error bars the 85% confidence intervals. Results support contact-end resistance measurements and show that the resistance in both c-GST and a-GST are dominated by the contacts for thin devices.

S3. Steady state *I-V* measurements, additional data

Most previous work which has sought to match between *I-V* measurements of a-GST and the current transport mechanisms have assumed a uniform electric field, $E \approx V/d$. However, here we show that the depletion approximation is more appropriate. We use Sentaurus TCAD simulations, with the thermionic emission model for current transport and the Schottky barrier height obtained from measurements (Figure 5f). Equilibrium and biased states were simulated by solving drift-diffusion equations coupled with Poisson's equation and Fermi-Dirac statistics.

We show the resulting electric field extracted for a defect concentration of 10^{20} cm^{-3} along a vertical cutline between the bottom and top electrodes, near the contact (up to a distance of 10 nm). The simulated electric field is presented along with the depletion approximation (in its relevant region) and the uniform field estimation, at a voltage of V_A=0.4 V, applied to the bottom electrode. We can see that the uniform field estimation is not sufficient to describe the electric field and that the depletion approximation is much more appropriate (Supporting Figure S2a).

The fitting parameters used in section 'Thermionic Emission (Schottky)' of Results and Discussion, N_T, the ionized trap density, and ε, the dielectric constant, are in the expected range (N_T~10^{20}cm^{-3}, ε~10).[7] Moreover, in equation (5), we included a built-in voltage (V_{BI}) of ~130 meV, extracted from the ultraviolet photoelectron spectroscopy (UPS) measurements.

Additionally, we performed ellipsometry measurements on a sputtered 50-nm a-GST blanket film, in order to confirm that the optical properties of thin films do not differ from thicker films. The obtained values, shown in Supporting Figure S2b, match the literature and validate the approximate value of the dielectric constant used.[7,8]

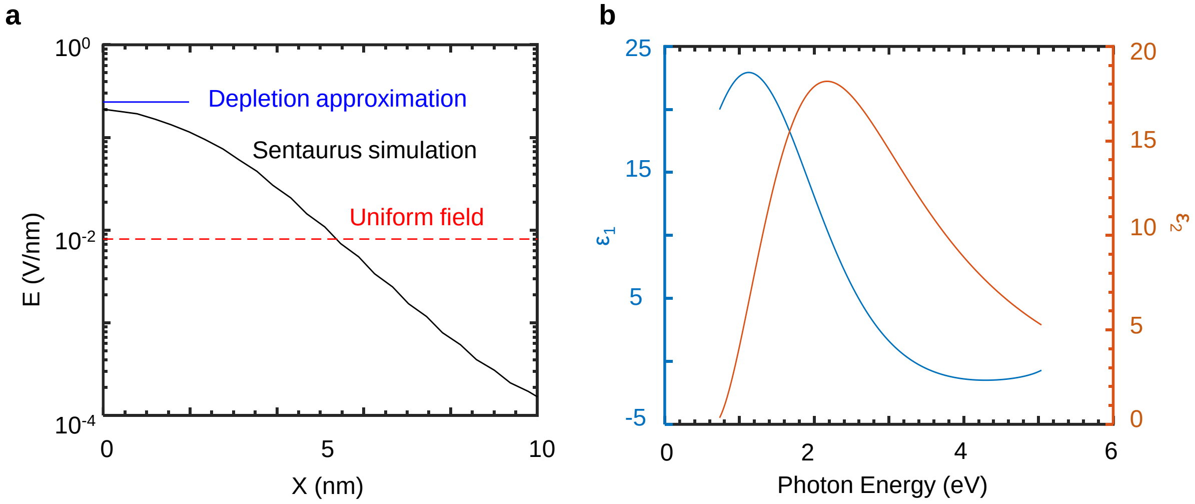

Fig. S2. (a) Comparison between electric field approximations and the Sentaurus TCAD simulation. Results show that the depletion approximation better represents the electric field in the vicinity of the interface. (b) Dielectric function of sputtered 50-nm a-GST substrate. Values match literature.[8]

Next, we show that even without the TLM measurements, the thermionic emission (Schottky) mechanism is more appropriate to describe the steady-state *I-V* data, measured for different ambient temperatures. Our measurements show a better fit to the thermionic emission expression than the Poole-Frenkel (PF) model, as can be seen visually in Supporting Figure S3.[9,10] For instance, at high temperatures the thermionic emission fits are separable, whereas the PF fits intertwine for different read voltages. Additionally, the calculated root-mean-square deviation (RMSE) of the FP fit is at least 0.026 higher than that of the thermionic emission fit, for all measured voltages. We conclude, as shown in the main text, that for thin a-GST films thermionic emission at the contacts is the dominant conduction mechanism.

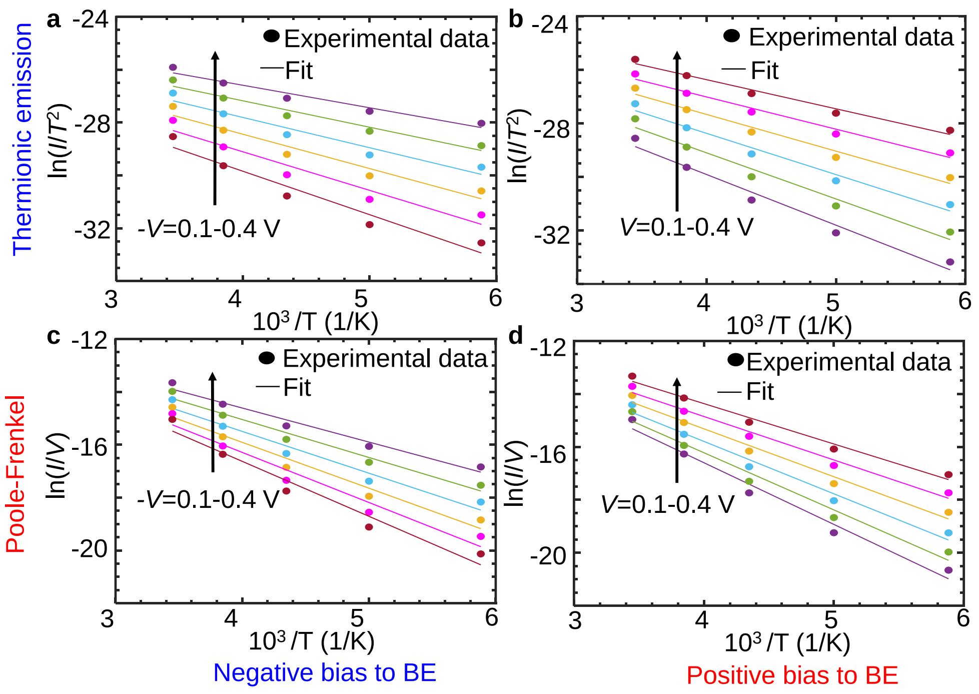

Fig. S3. Thermionic emission vs Poole-Frenkel fit. Thermion emission fit for (a) negative (b) positive bias to BE. Poole-Frenkel fit for (c) negative (d) positive bias to BE. Thermionic emission shows a better fit.

S4. Ultraviolet photoelectron spectroscopy, additional data

In this work we use pump/probe ultraviolet photoelectron spectroscopy (UPS). This was carried out at a photon energy of 26.35 eV using the high harmonics (17th) from an amplified Ti:sapphire laser system. In addition to direct photoelectron spectra collected from the ground state of the GST, time correlated photoexcitation of the samples was used to flatten the bands of the GST. Band bending was determined by comparing the energetic difference between the pumped and un-pumped spectra. This system has been described in detail previously.[11]

The Fermi level of the system was determined from spectra collected from an Al film deposited on the sample after experiments were carried out. For all UPS spectra shown in this paper, binding energies of spectral features are referenced to the Fermi level (0 eV binding energy). GST samples were dipped in dilute HF (10:1 distilled water) to remove oxides and a mild anneal (100˚C) was used to drive off atmospheric water physiosorbed prior to insertion into the vacuum system. Anneals (10s duration following stabilization of temperature) of the GST were carried out in vacuum (base pressure ~$3x10^{-10}$ torr) using an optical pyrometer to monitor temperature. Barrier heights

were extracted from the photoelectron spectra by utilizing the measured location of the Fermi level, relative to the valence band edge of the GST samples.

We note that the UPS measurements show the average barrier height,[12] while when performing electrical measurements, the extracted barrier height is the minimal one,[13] explaining the small difference in barrier height values obtained in the main text.

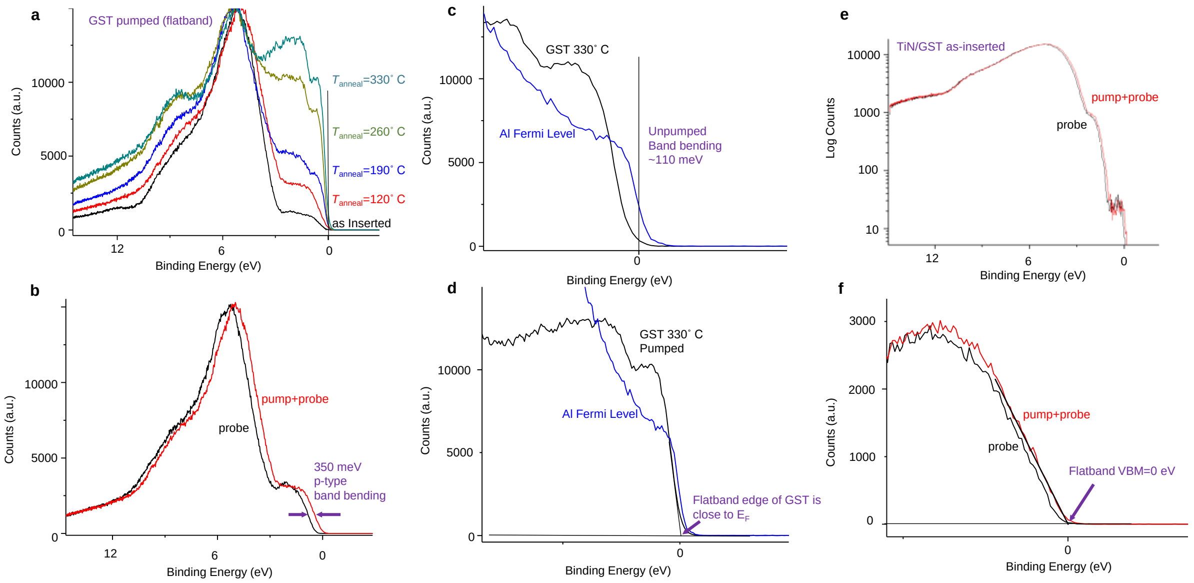

Fig. S4. UPS measurement data (a) GST anneal series. The 0 energy corresponds to the Fermi level. (b) Comparison of probe only (black) and pump+probe (red) for GST annealed to 120˚C for 10s. The upward shift of the pumped spectrum relative to the probe only indicates the p-type nature of the GST. (c) Comparison of probe only GST annealed to 330˚C for 10s (black) and location of the Fermi edge of Al (blue). (d) Comparison of pump+probe spectrum of flat band GST (black) with Al Fermi edge (blue). (e) Comparison of as-inserted TiN/GST probe only (black) and pump+probe (red). (f) Blow-up of probe only (black) and pump+probe (red) spectra of C-GST annealed to 120˚C. From here we extract Schottky barrier heights: a-GST/TiN Schottky barrier=243 meV; c-GST/TiN Schottky barrier=80 meV; a-GST/c-GST Schottky barrier=34 meV (following 330˚C anneal);

S5. Interface dominated multi-level conduction in mushroom-type cells

In this section we explain conduction in multi-level mushroom-type cells, with the understanding that conduction in thin GST devices is dominated by interfaces. Figure S5 schematically illustrates partial crystallization of a mushroom PCM cell. Because crystallization in GST is nucleation dominated,[14] with increasing pulses additional nucleation sites are present until percolation paths are formed (shown in blue in Supporting Figure S5c). These paths' resistance forms in parallel to the existing interface resistance (either top or bottom), and the equivalent intermediate resistance can be explained as the combination of the two.

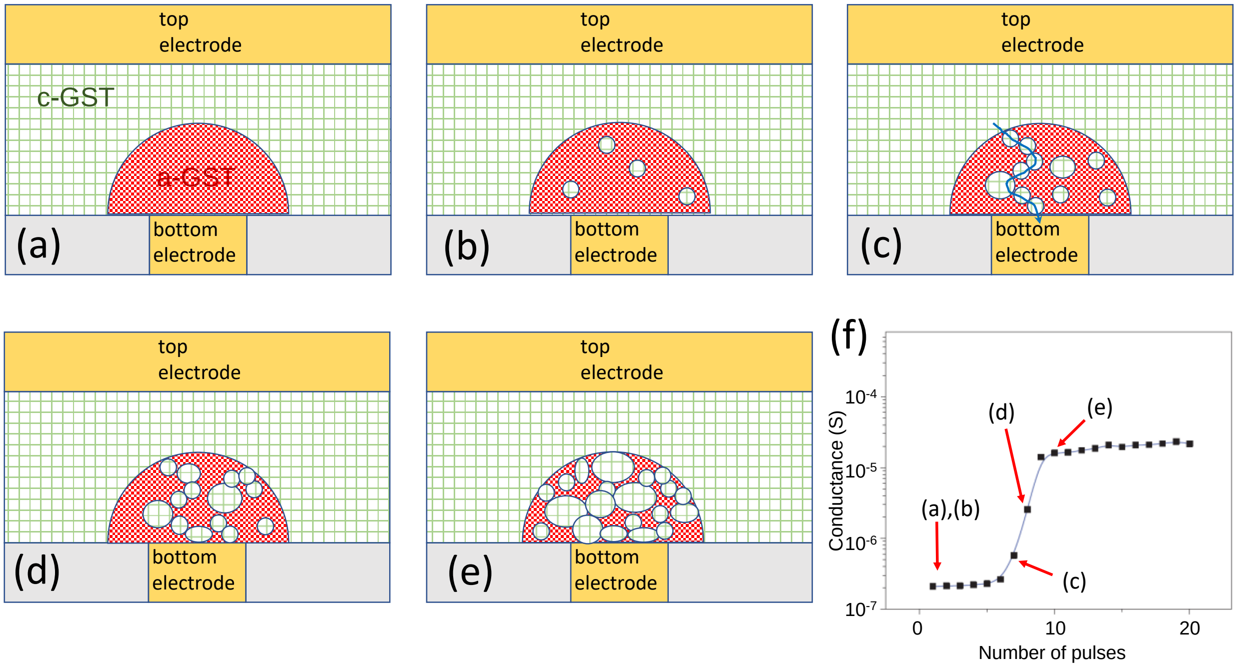

Fig. S5. Schematic illustration of nucleation-dominated crystallization of multi-level mushroom-type cell, based on results presented by T. Tuma *et al*.[15] (a)-(e) Illustration of changes is a-GST regime due to partial crystallization, from low conductance to high conductance. Gradually, more and more nucleation sites are present. (f) conductance of mushroom-type cell as a function of number of pulses for the different states illustrated in (a)-(e) [15]

Furthermore, Supporting Figure S6 shows electrical measurements of a mushroom-type cell for different intermediate resistances. For low intermediate resistances (such as the one illustrated in Supporting Figure S5e) the percolation paths are more dominant, and a more symmetrical behavior is observed. However, clear asymmetric behavior can be noticed for high resistances. This is likely

due to the asymmetric cell geometry and the different interface areas (the polarity of the applied voltage defines which interface is in reverse bias and dominant). This result, for mushroom-type cells, is consistent with our findings, that the resistance for thin devices is dominated by interfaces.

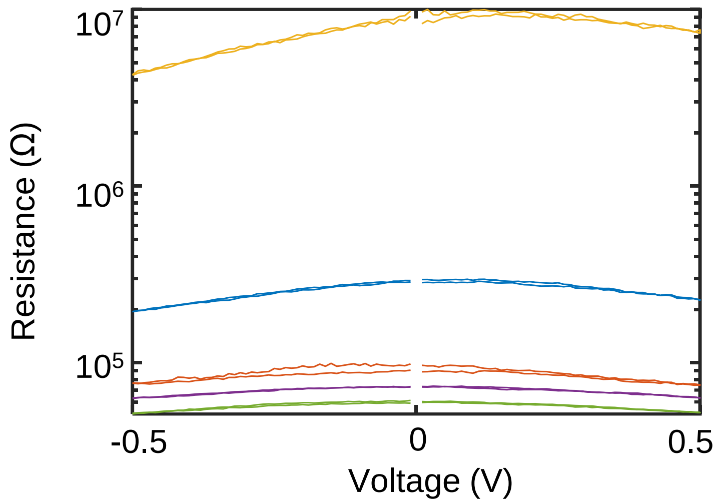

Fig. S6. Resistance vs read voltage measurements of mushroom-type cell, with multiple intermediate states. The asymmetric behavior of high resistances indicates the importance of interfaces when evaluating intermediate states.

S6. Schottky barrier height drift, additional data

The following section demonstrates that the subtle trend observed in Figure 7b is an artifact of data processing procedure. We relate this to the time of the first measurement after reset as a single value, t_0, yet that is not the case, as shown in the inset of Figure 1c and Supporting Figure S7a. The reason is that the resistance drifts even during the measurement of the first *I-V* sweep. Therefore, for decreasing voltages (marked in blue in Supporting Figure S7a) the current also decreases and the trend is as shown in Supporting Figure S7c. The data shown in Figure 7b and Supporting Figure S7b correspond to increasing voltages (marked in red in Supporting Figure S7a) and therefore show the opposite trend. In order to overcome this issue, the change in the barrier height is obtained by averaging over all measured voltages of a single sweep. There is an inherent trade-off between fast measurements and accuracy of low-noise current measurement.

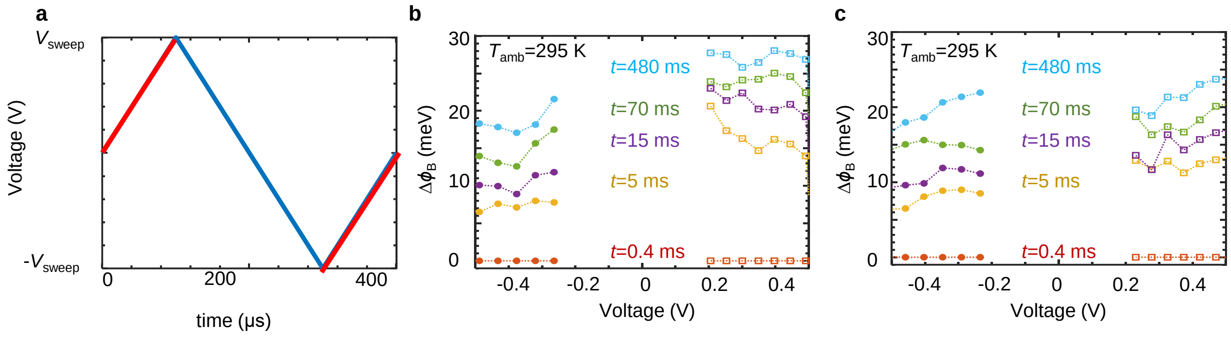

Fig. S7. Extraction of the barrier height for varying drift time following a reset pulse with clarification regarding t_0 (a) single *I-V* sweep in linear scale. (b) extraction of the barrier height for decreasing voltages as shown in blue in (a). (c) Extraction of the barrier height for increasing voltages as shown in red in (a).

S7. Transmission electron microscopy images

The following bright field scanning transmission electron microscope (BF-STEM) images show an a-GST device from the same die used for the electrical measurements in our work. We reset the device to a high resistance of 1.5 MΩ. Marked in yellow in Supporting Figure S8b is the amorphous region, which has an estimated area of 18000 nm^2 and reaches both the top (TE) and bottom (BE) electrodes. This amorphous area can also be seen in Supporting Figure S8c in the diffractogram from the high-resolution BF-STEM image. The energy dispersive X-ray spectroscopy (EDX) elemental maps of the device structure are shown in Supporting Figure S9.

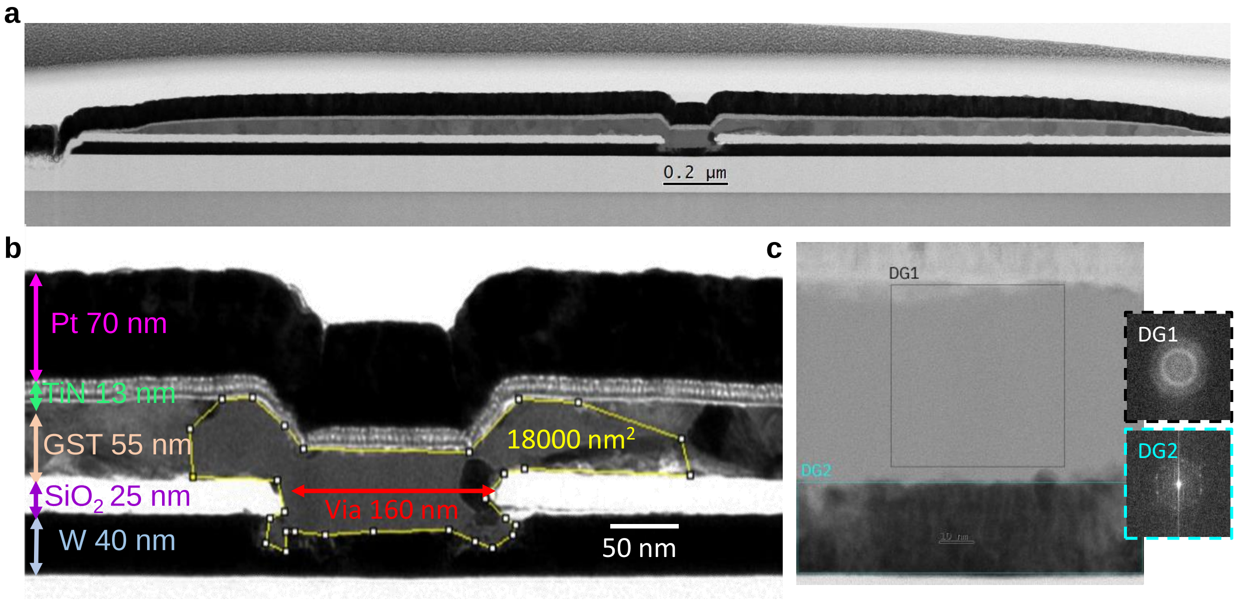

Fig. S8. BF-STEM image of device reset to 1.5 MΩ. GST is ~50 nm thick between tungsten (W) and titanium nitride (TiN) electrodes. (a) Wide view of the device, at the center is confined cell (b) Confined cell with dimensions labeled. The amorphous region is marked in yellow and has an area of approximately 18000 nm^2 (c) Diffractograms from high resolution BF-STEM image not capturing crystalline features (DG1) and showing crystalline features in tungsten (DG2).

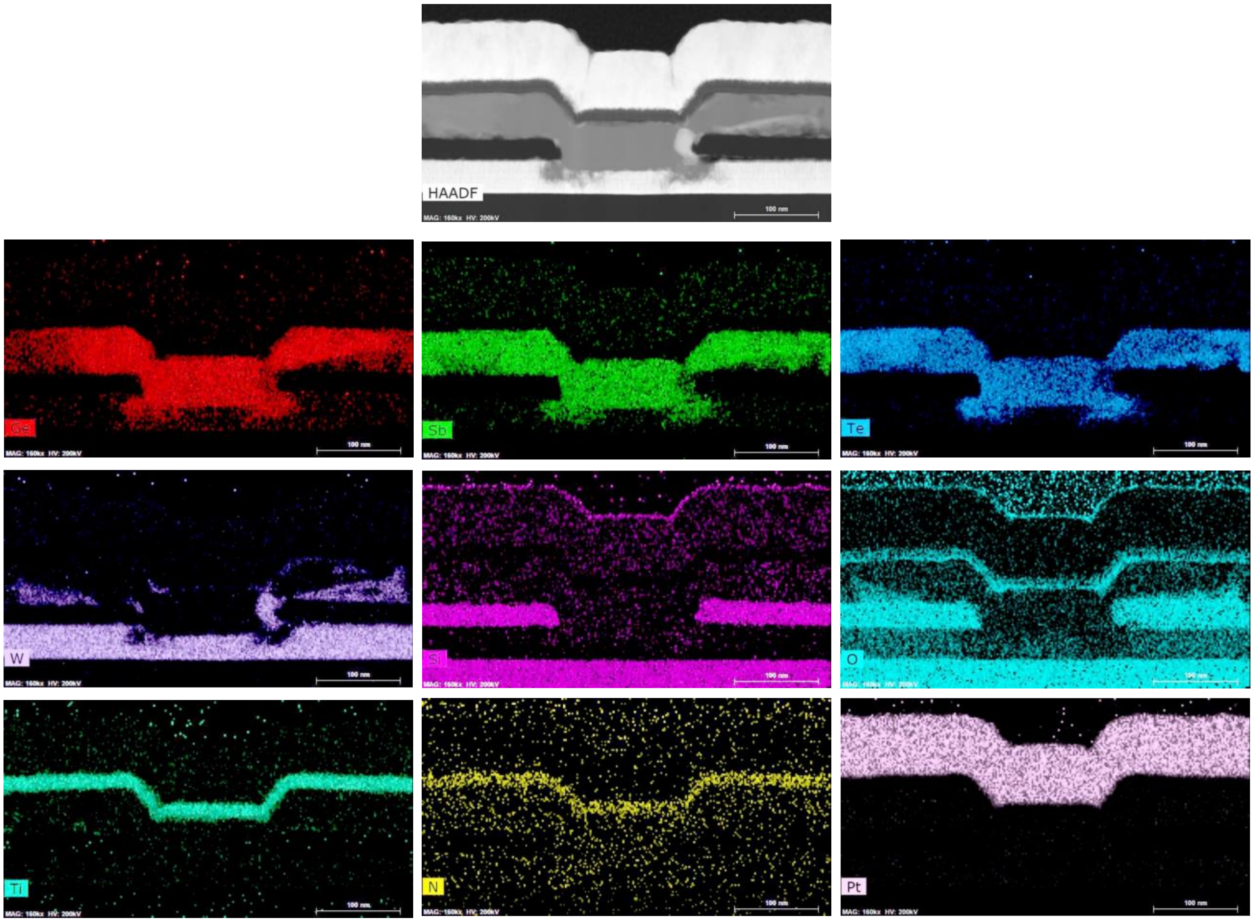

Fig. S9. High angle annular dark field (HAADF) STEM image and energy dispersive X-ray spectroscopy (EDX) elemental maps showing the device elemental distribution.

Supporting Information References:

(1) Raoux, S.; Cohen, G.; Shelby, R.; Cheng, H.-Y.; Jordan-Sweet, J. Amorphization of Crystalline Phase Change Material by Ion Implantation. In *Materials Research Society (MRS) Online Proceedings Library*; 2010. https://doi.org/10.1557/PROC-1251-H02-06

(2) Privitera, S. M. S.; Rimini, E. Ion Beam Irradiation of Phase Change Materials: A Route to Material Properties Investigation and Engineering. *Materials Science in Semiconductor Processing*. 2021. https://doi.org/10.1016/j.mssp.2021.106087.

(3) Maria, S.; Privitera, S. Ion Implantation in Phase Change Ge2Sb2Te5 Thin Films for Non Volatile Memory Applications. In *Ion Implantation*; InTech, 2012; pp 129–152.

(4) Savransky, S. D.; Karpov, I. V. Investigation of SET and RESET States Resistance in Ohmic Regime for Phase-Change Memory. *Materials Research Society (MRS)*; 2008. https://doi.org/10.1557/PROC-1072-G06-09

(5) Schroder, D. K. Contact Resistance and Schottky Barriers. In *Semiconductor Material and Device Characterization*; Wiley-Interscience, 2006; pp 127–184.

(6) Ber, E.; Grady, R. W.; Pop, E.; Yalon, E. Uncovering the Different Components of Contact Resistance to Atomically Thin Semiconductors. *Adv Electron Mater* 2023, *9* (6). https://doi.org/10.1002/aelm.202201342.

(7) Chen, C.; Jost, P.; Volker, H.; Kaminski, M.; Wirtssohn, M.; Engelmann, U.; Krüger, K.; Schlich, F.; Schlockermann, C.; Lobo, R. P. S. M.; Wuttig, M. Dielectric Properties of Amorphous Phase-Change Materials. *Phys Rev B* 2017, *95* (9). https://doi.org/10.1103/PhysRevB.95.094111.

(8) Park, J. W.; Eom, S. H.; Lee, H.; Da Silva, J. L. F.; Kang, Y. S.; Lee, T. Y.; Khang, Y. H. Optical Properties of Pseudobinary GeTe, Ge2 Sb2 Te5, GeSb2 Te4, GeSb4 Te7, and Sb2 Te3 from Ellipsometry and Density Functional Theory. *Phys Rev B Condens Matter Mater Phys* 2009, *80* (11). https://doi.org/10.1103/PhysRevB.80.115209.

(9) Sze, S. M.; Li, Y.; Ng, K. K. *Physics of Semiconductor Devices*; Wiley-Interscience, 2006.

(10) Kao, K. C. *Dielectric Phenomena in Solids with Emphasis on Physical Concepts of Electronic Processes*; Elsevier Academic Press, 2004.

(11) Haight, R. Electronic Structure of Technologically Important Interfaces and Heterostructures. *MRS Communications*. 2020, pp 529–537. https://doi.org/10.1557/mrc.2020.63.

(12) Whitten, J. E. Ultraviolet Photoelectron Spectroscopy: Practical Aspects and Best Practices. *Applied Surface Science Advances* 2023, *13*. https://doi.org/10.1016/j.apsadv.2023.100384.

(13) Werner, J. H.; Güttler, H. H. Barrier Inhomogeneities at Schottky Contacts. *J Appl Phys* 1991, *69* (3), 1522–1533. https://doi.org/10.1063/1.347243.

(14) Raoux, S.; Wuttig, M. *Phase Change Materials*; Raoux, S., Wuttig, M., Eds.; Springer US: Boston, MA, 2009. https://doi.org/10.1007/978-0-387-84874-7.

(15) Tuma, T.; Pantazi, A.; Le Gallo, M.; Sebastian, A.; Eleftheriou, E. Stochastic Phase-Change Neurons. *Nat Nanotechnol* 2016, *11* (8), 693–699. https://doi.org/10.1038/nnano.2016.70.
